# Psychiatric Manifestations of Iron Deficiency Anemia-A Literature Review

**DOI:** 10.1192/j.eurpsy.2023.560

**Published:** 2023-07-19

**Authors:** H. Arshad, A. Arshad, M. Y. Hafiz, G. Muhammad, S. Khatri, F. Arain

**Affiliations:** ^1^Psychiatry; ^2^Medicine, Jinnah Sindh Medical University; ^3^Psychiatry, Aga Khan University Hospital; ^4^Medicine, Karachi Medical And Dental College, Karachi, Pakistan; ^5^Psychiatry, Ocean Medical Center; ^6^Psychiatry, Rutgers New Jersey School of Medicine, New Jersey, United States

## Abstract

**Introduction:**

Anemia due to iron deficiency is a highly prevalent medical condition in women and children. Iron deficiency presents with fatigue, low mood, anxiety, restlessness, palpitations, and headache. Poor nutritional intake can be the reason of iron deficiency in underprivileged populations. It can lead to behavioral symptoms that can manifest as chronic psychiatric ailments.

**Objectives:**

Our objective is to consolidate manifestations of iron deficiency anemia concerning psychiatric ailments. We will figure out if it impacts the severity of psychiatric symptoms. We aim to find out if there are any underlying factors that impact the correlation of iron deficiency with psychiatric disorders like depression, anxiety, sleep disorders, and restless leg syndrome.

**Methods:**

Detailed literature review conducted using PUBMED, OVID, GOOGLE SCHOLAR with the search terminologies [iron] OR [sleep disorders] OR [depression] OR[deficiency] OR [anxiety] OR [ADHD] OR [VITAMINS] OR[PICA] OR [CHILDREN] OR [women] OR [antidepressants] OR [sleep medicine] OR [antipsychotics] that yielded 150 results that were narrowed down to be focused on our research area. Inclusion criteria included studies with participants with iron deficiency anemia regardless of age group, gender, economic and social background. Exclusion criteria included patients with normal hemoglobin levels.

**Results:**

Results yielded a positive impact of treating iron deficiency anemia in patients with psychiatric ailments. The symptoms of low mood, fatigue, anxiety, anhedonia, and sleeplessness get better as iron deficiency improves. According to the search, some physicians misdiagnose iron deficiency as depression. Antidepressants were found to be working better when added with iron supplements. Restlessness and palpitations can also be the manifestations of iron deficiency. Patients with underlying iron deficiency are more predisposed to developing psychiatric disorders. According to published data, restless leg syndrome was found to be associated with iron deficiency. Some psychiatric drugs can lead to iron deficiency and can provoke underlying iron deficiency even more. Iron deficiency impacts memory areas of the brain like the hippocampus and prefrontal cortex.

**Conclusions:**

It is much needed more than ever before that proper consideration to the diagnosis of iron deficiency anemia must be given with the assistance of predesigned guidelines. Misdiagnosis of iron deficiency anemia as a psychiatric disorder can be misleading toward the insidious usage of psychiatric medications. Proper attention must be provided to this neglected area so that management of iron deficiency is tailored in the right direction and it is diagnosed at less severe stages. It will be helpful for general physicians and practicing psychiatrists in the field.

Keywords: Iron deficiency, Psychiatric Disorders, Anxiety, depression.

**Disclosure of Interest:**

None Declared

